# Supplementary figures and images for: Molecular mechanism of proton-coupled ligand translocation by the bacterial efflux pump EmrE
Source: PLoS Comput Biol. 2021 Oct 6;17(10):e1009454. doi: 10.1371/journal.pcbi.1009454 (PMC8523053; doi:10.1371/journal.pcbi.1009454)

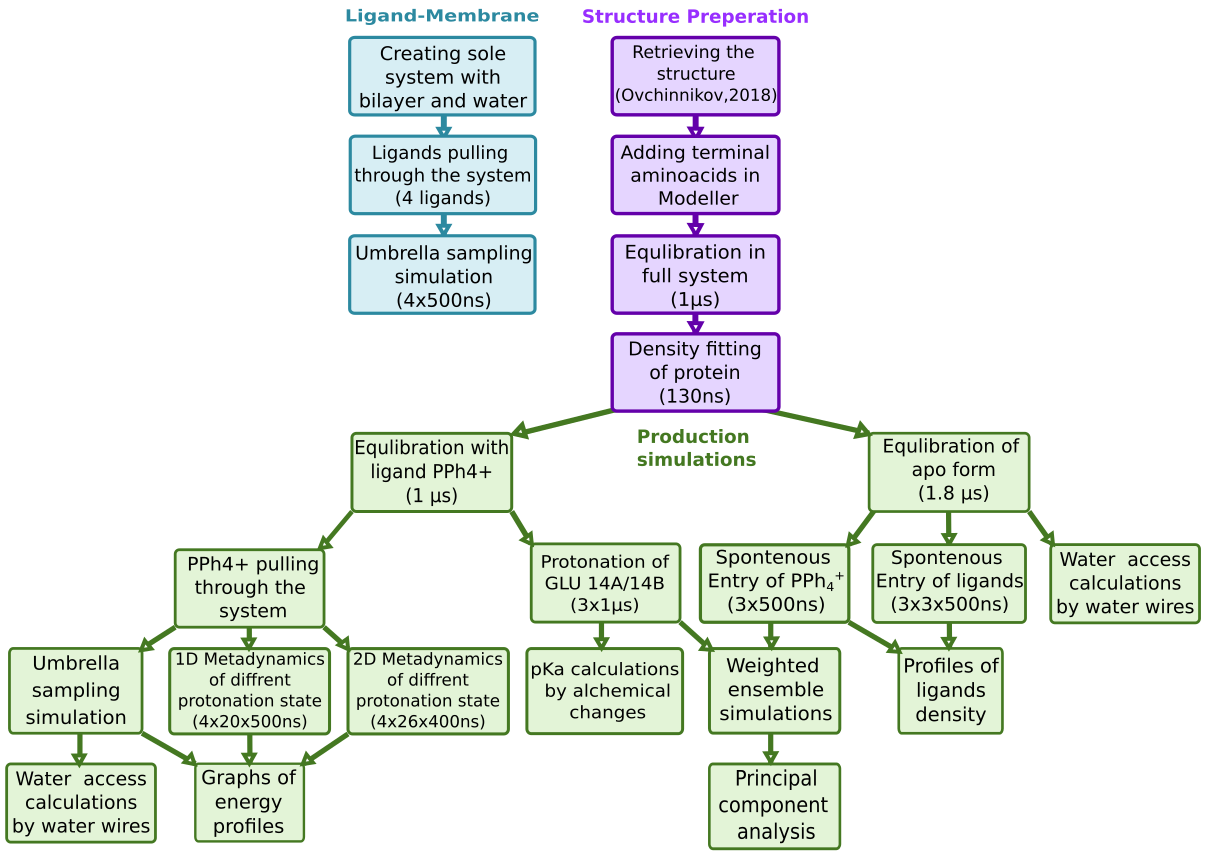

Supplement: S1 Fig — See Methods for the details of all approaches used. (TIF) [file pcbi.1009454.s001.tif]

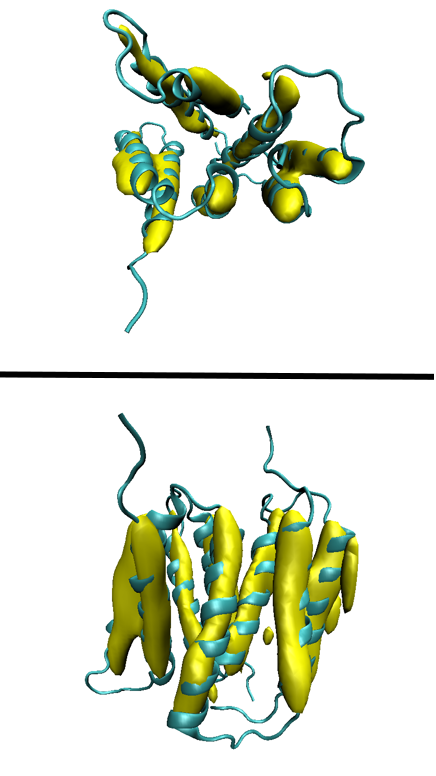

Supplement: S2 Fig — Using an early implementation of the cryo-EM module in the Gromacs package, we set the densfit-sigma parameter to 0.45 nm and densfit-k changed linearly from 0 to 10000 over 5 ns and remained at 10000 for the subsequent 95 ns. In the implementation, the “forward” model of the electron density is created by Gaussian-smoothing the atomic structure, and the forces are then applied to minimize the cross correlation between the simulated and actual density. This method allowed to obtain a structure with a proper spacing and orientation of helices, corresponding to the PDB entry 3B5D. (TIF) [file pcbi.1009454.s002.tif]

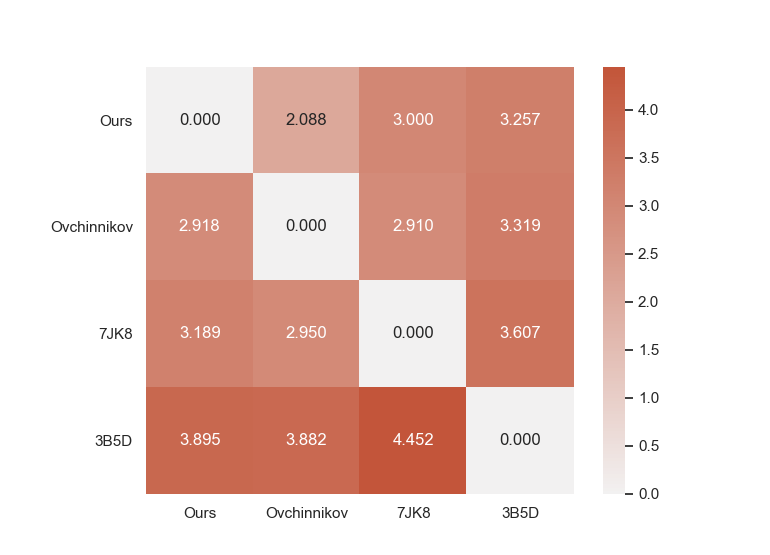

Supplement: S3 Fig — The lower part of the matrix contains RMSD values based on the full protein (except for the flexible C- and N-termini), while the upper part shows the corresponding values with the alpha carbons of the loops omitted. Legend: Ovchinnikov, the starting structure for this study taken from [28]; 7JK8, the most recent (2021) NMR structure [29]; 3B5D, the CA-only low-resolution structure that was first reported in 2007 [26]. (TIF) [file pcbi.1009454.s003.tif]

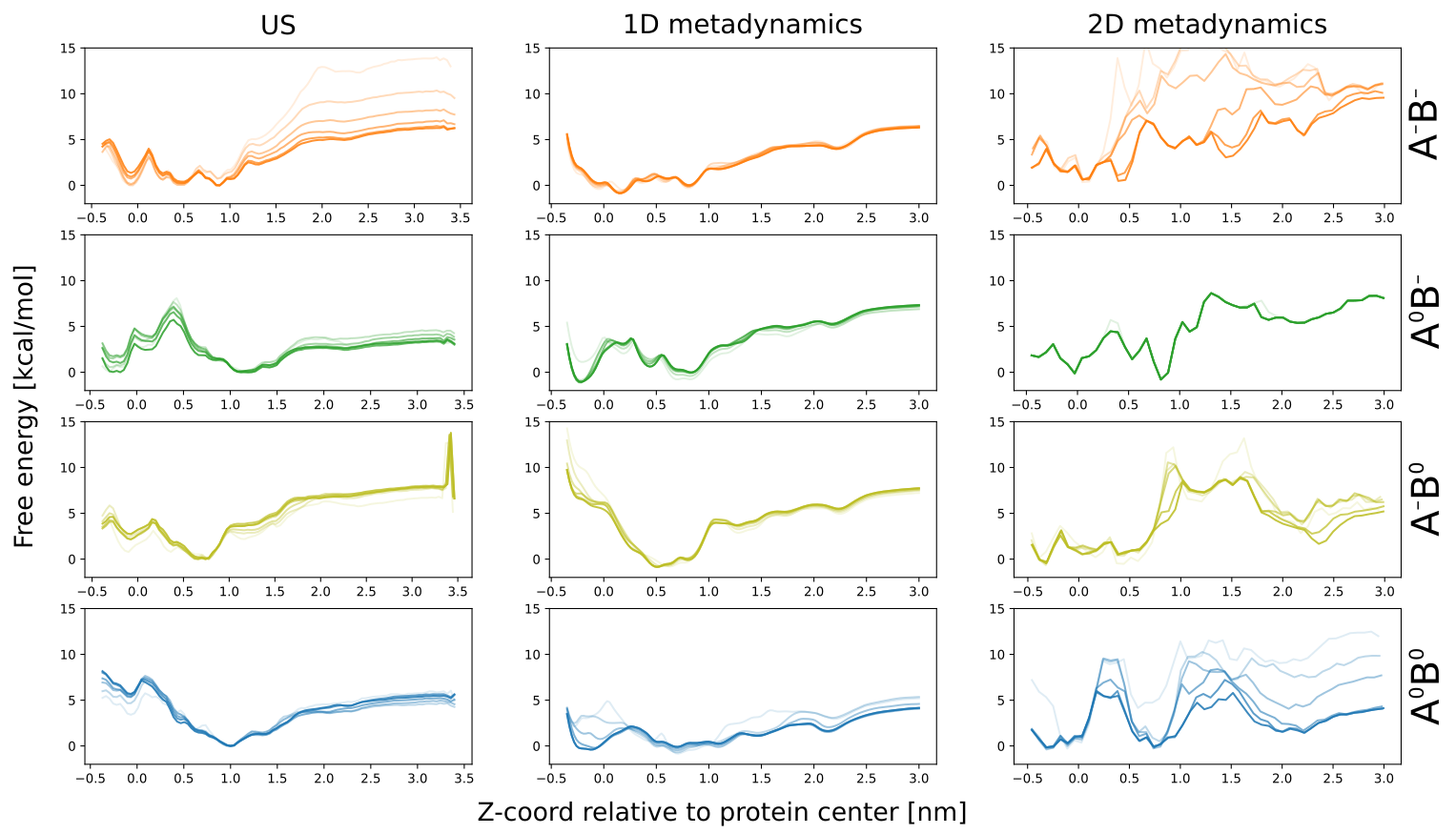

Supplement: S4 Fig — For the convergence analysis, data was divided into 6 batches and calculations were performed on cumulative datasets (batch 1, batch 1+2, …). (TIF) [file pcbi.1009454.s004.tif]

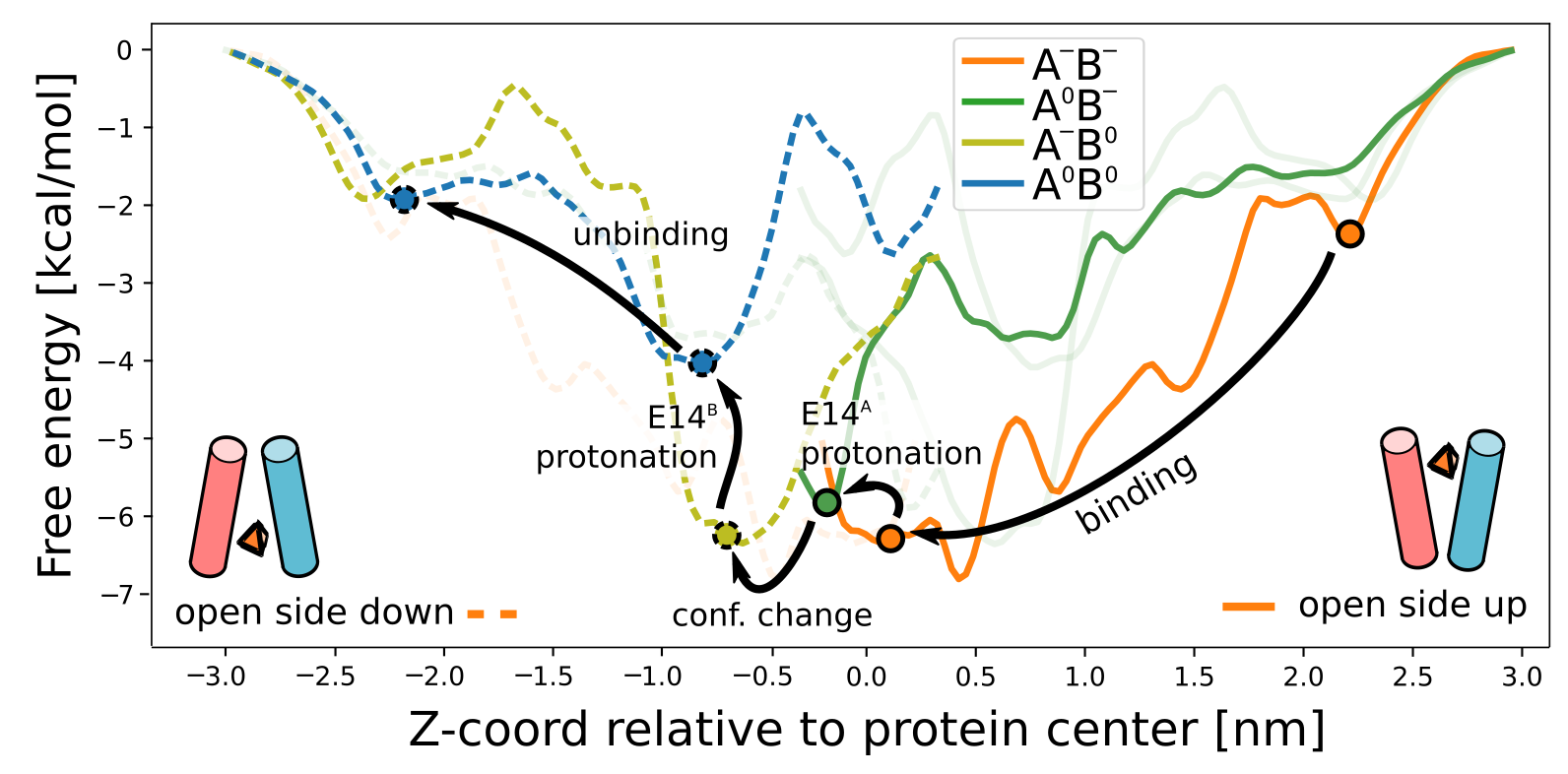

Supplement: S5 Fig — This pathway is relatively more plausible under low cytoplasmic pH, and may represent an adaptation to variable pH conditions. (TIF) [file pcbi.1009454.s005.tif]

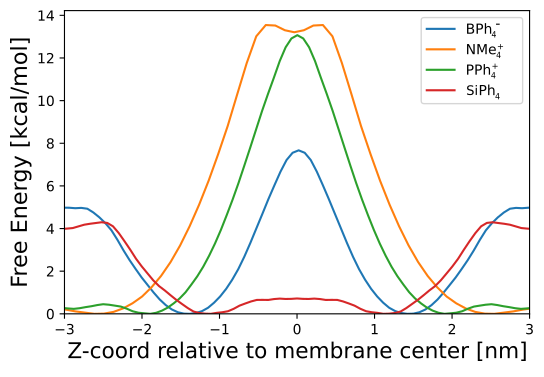

Supplement: S6 Fig — The membrane was generated using the CHARMM-GUI webserver. The umbrella sampling protocol was applied to four different ligands (aromatic: cationic PPh4+, neutral SiPh4, anionic BPh4−, and non-aromatic hydrophobic cation NMe4+) to create free energy profiles as a function of the z coordinate. The profiles indicate that charged ligands preferentially reside in the aqueous phase near the bilayer surface, the anionic ligand resides in the headgroup region and can likely pass through the membrane in a spontaneous manner, while the neutral tetraphenylsilane shows sizeable affinity for the bilayer interior. (TIF) [file pcbi.1009454.s006.tif]

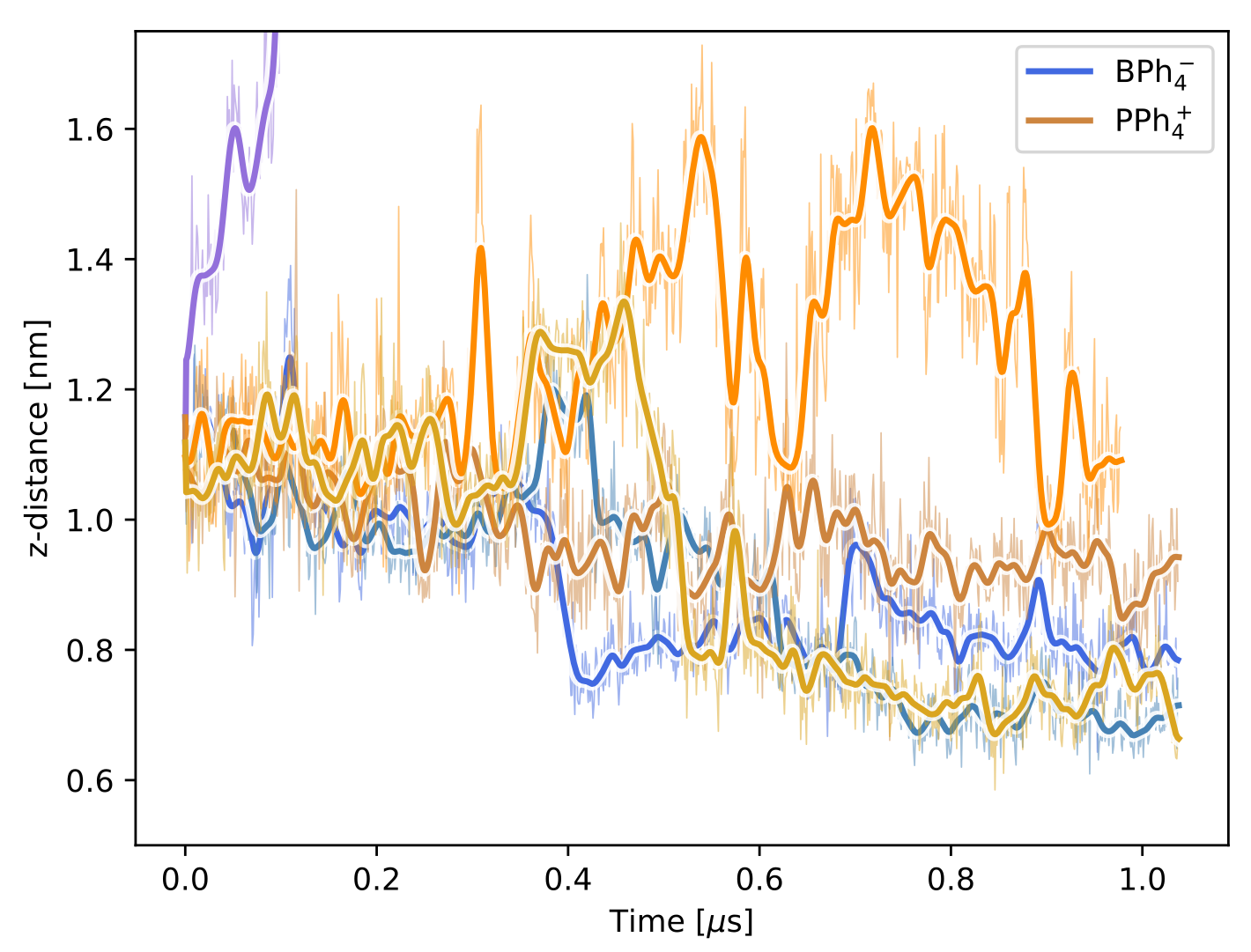

Supplement: S7 Fig — 3-channel ligand structures (distance from to the binding site between 1.0 and 1.2 nm) were taken from the equilibrium simulation and six simulations of 1 μs each were performed for 3 systems with PPh4+, and 3 for BPh4−. The performed simulations were run as “as it is” or by changing the charges while maintaining the geometry. Of the 3 systems containing PPh4+ (yellow / orange / brown lines), all remained stably related to the channel entry, mainly going deeper (up to 0.8 nm) into the channel. At the same time, the least bound BPh4− dissociated in one of the systems (purple line), while the other two progressed deeper into the binding channel, as did their cationic counterparts (blue / turquoise lines). (TIF) [file pcbi.1009454.s007.tif]

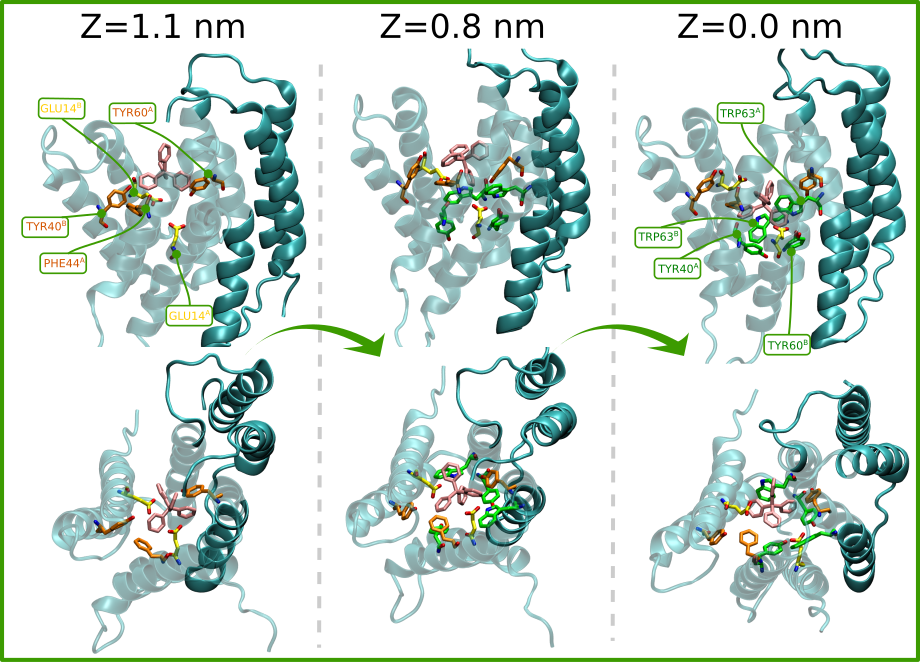

Supplement: S8 Fig — Simulations obtained from Westpa provide an insight into the key amino acids involved in the ligand entrance into the binding site, here visualized at three crucial stages (Z = 1.1 / 0.8 / 0.0) where the ligand dwells due to the presence of free energy barriers. The amino acids actively involved in the transport mechanism are shown: E14, Y60, Y40, F44 and W63. (TIF) [file pcbi.1009454.s008.tif]

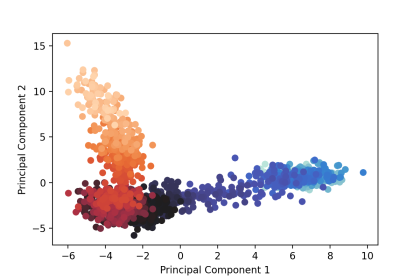

Supplement: S9 Fig — The short trajectory from Westpa was merged with a follow-up 100 ns equilibration. (TIF) [file pcbi.1009454.s009.tif]

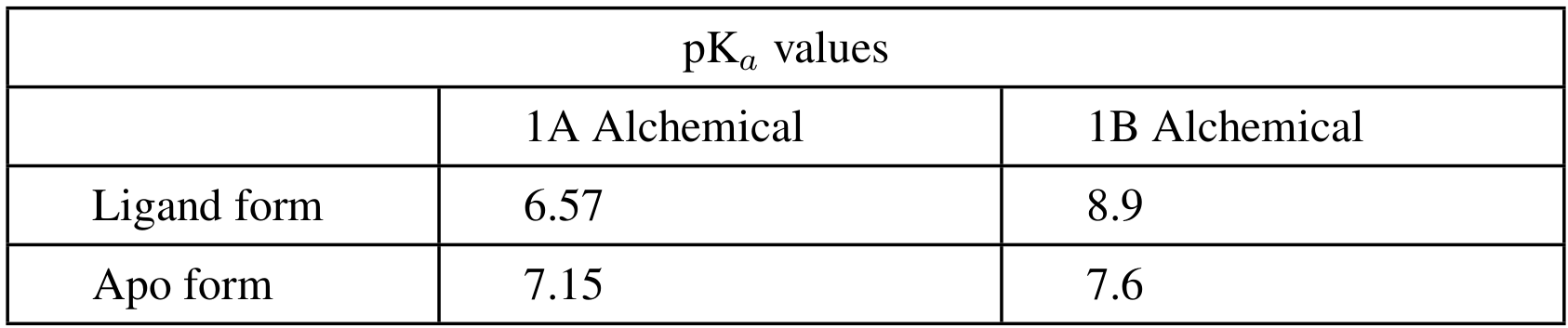

Supplement: S1 Table — The table shows the pKa values obtained for a system with the ligand in the active site (Ligand-bound) and without it (Apo form). The vertical column labels (E14A/E14B) denote simulations in which a proton was alchemically exchanged between either E14A or E14B embedded in the protein and a capped free GLU residue placed in the water phase, as described in the Methods. (TIF) [file pcbi.1009454.s010.tif]
