# Supplementary material for: Molecular mechanism of proton-coupled ligand translocation by the bacterial efflux pump EmrE
Source: PLoS Comput Biol. 2021 Oct 6;17(10):e1009454. doi: 10.1371/journal.pcbi.1009454 (PMC8523053; doi:10.1371/journal.pcbi.1009454)
Supplement: Attachment — Submitted filename: Responses.pdf [file pcbi.1009454.s014.pdf]

We thank the reviewers for their valuable suggestions and insightful comments that helped improve the quality of our manuscript. Below, we address each concern raised by the reviewers.

Reviewer 1

1. Why were both umbrella sampling and metadynamics used for free-energy calculations? And how were they combined? I don't think that you can just average distinct free-energy profiles. The authors should provide a mathematically rigorous approach for combining (if they still want to do so) and/or provide individual profiles in the SI.

   The idea of averaging over multiple independent free energy profiles (originally for fluctuations in metadynamics simulations) was put forward in an article by Laio and Gervasio (10.1088/0034-4885/71/12/126601).
   From experimental data, it was known that each protonation of the central glutamates decreases the affinity for the ligand, so any plausible free energy profiles had to follow this qualitative ordering of affinities. However, even with very extensive sampling individual free energy runs converged to profiles that did not meet this criterion individually, failing to equilibrate along slower degrees of freedom. For this reason, we restarted the calculations from scratch twice until an approach combining all available data met the experimental restraint.
   We now include the individual profiles in Fig. S4.

2. Related, the authors should provide plots indicating the convergence of the free-energy profiles.

   Convergence is now shown in Fig. S4 as well.

3. I find Figures 2C and 2D too busy and difficult to interpret. I don't think the faded profiles, for example, help. Can the authors find a way to simplify the presentation?

   We moved the panel 2D to SI (now Fig. S5) to make Fig. 2 cleaner and extended the discussion of panel 2C in the caption. We also reduced the amount of labels in Fig. 2C. The faded profiles are meant to remind that the plot was duplicated through reflection to represent the free energy profiles after the conformational change, and after consulting with people not involved in the study we decided to (a) keep them to avoid confusion but also (b) state that explicitly in the caption.

4. Can the authors propose new experiments that could test some of their conclusions?

   We remarked in the Discussion that our proposed translocation pathway has non-trivial implications for inhibitor design, suggesting that only asymmetric bivalent ligands would physically block the translocation. Residue-specific conclusions can be tested in mutagenesis studies – for example, replacing W63 and/or M21 might disrupt the water-tight seal in the apo form leading to unproductive proton leakage, while disrupting the surface-exposed clusters of aromatic residues might affect transfer rates in a concentration-dependent manner (due to a lack of initial low-affinity binding sites targeting the ligand to the binding site).

Reviewer 2

1. In the introduction, it is stated that "To date, the transport mechanism of EmrE has not been unambiguously established on the atomic level, nor has a high-quality structure been obtained experimentally, a common issue among small flexible proteins". However, a new NMR based structure was released earlier this year with PDBID 7JK8 (reference 46). I fully realize that this project was likely started before this structure was made available, but the text needs to be changed to reflect this reality, and the appropriate comparisons need to be made to provide the reader with the full context for these results.

   Indeed, this remark was originally written well before the new structure was published,

and while NMR is traditionally considered a lower-resolution method than X-ray or cryo-EM, the new structure definitely merits a mention earlier in the Introduction (now included in the text).

2. Where did you obtain the starting PDB structure? The Ovchinnikov model isn't published alongside their paper, and so a reader can't really assess for themselves how big the structural changes you imposed by refitting to CryoEM are.

   The PNAS article by Ovchinnikov et al. is accompanied by a Mendeley Dataset (data.mendeley.com/datasets/3pvz4hytfd/2) that contains the optimized structure of EmrE.

3. It would be interesting to know how different the two models are, especially when compared to the recent NMR structure. RMSDs would be easy to calculate, and can help to assess how similar or different the models are from one another. It would also be valuable for the wider community if the EmrE model generated were to be released along with the SI.

   We calculated the relevant metrics and compiled them in Fig. S3. Our optimized EmrE model is now available at `https://gitlab.com/KomBioMol/emre-setup`.

4. Figure 2 is full of information! Possibly too much to be put into one figure. Particularly C and D are hard to understand, since it looks like there are 3 binding events under consideration, when it is clear that only 1 TPP binds. In panel D, E14A protonates twice! I think in this case, I might demote C and D to SI panels, and instead use simplified energy level diagrams like you might see in QM papers (eg. Fig. 5 from https://www.mdpi.com/2073-4344/9/11/887/htm, which happened to be the first one Google images came up with) to highlight the alternative energy landscapes in TPP translocation.

   Following the suggestion, we moved panel D to SI (now Fig. S5) and cleaned up the labels. We agree that parts of the scheme are counterintuitive at first, since one has to follow three dynamical variables – the z-coordinate of the ligand, the protonation state and the conformational state. Even worse, the conformational change swaps residue labels (in terms of e.g. pKas, A becomes B and vice versa). We decided to keep the existing representation in panel C to facilitate direct comparison with Fig. 2B (as originally intended), but added a more detailed explanation in the figure caption. We also refer the readers to Movie S1, one that was made exactly to visualize the process in a dynamic animation.

5. In Figure 3A, what were the parameters used to assess the presence or absence of a water wire? Typically, a geometric cutoff for both heavy-atom distance and angle linearity are used, but I can't find where they are specified. I'll take your word that Figure 3D shows what you say it does, but it is too small for a viewer to see the residues clearly.

   The h-bonds were classified using an acceptor-donor distance criterion of 0.35 nm and a donor-hydrogen-acceptor angle criterion of 120 degrees, as now specified in Methods. The concept behind Fig. 3D was indeed very hard to visualize in a 2D picture, so the reader is now encouraged to consult Movie S2 for a more robust visualization.

6. Methods notes: Please specify the GROMACS version used for simulation (e.g. 5.1, 2020, etc). Sometimes there are bugs that are discovered later, and not having the version number available makes it unclear to readers in the future if these simulations might have this bug present or not. I assume the protein and TPP+ parameters also come from the CHARMM36 family. Citations should be included for maximal clarity, and consider including the parameters used for TPP as an SI component.

   We have now added specific version information. We also deposited the optimized TPP+ parameters at `https://gitlab.com/KomBioMol/emre-setup`.

7. Do I understand correctly that the pKa calculations were carried out twice? Once moving the proton from an external glutamate to E14A, and once moving the proton from the

external glutamate to E14B? Experimentally, the pKas for both glutamates are known (abstract of reference 38), and while E14A in the apo form and liganded forms bracket the experimental values, the manuscript would be strengthened by actually making the comparison within the text. Qualitatively, the results I see in the table S1 agree with prior modeling studies that measured delta pKa (reference 27), identifying E14B as the site that has the higher pKa, and therefore is protonated more frequently. I would, however, take another look at the labeling surrounding table S1, since the labels are currently insufficient to clarify what was actually calculated.

The pKa calculations were performed separately for each monomer's glutamate, E14A and E14B, in both the ligand-bound and apo state, yielding a total of 4 calculations. Indeed, this is the qualitative result that we found, meant as a consistency check rather than new information. We agree that Table S1 wasn't sufficiently clear and so improved the presentation of pKa values in the revised version.

8. Combining pKa measurements here along with prior experiment, it seems clear that one of the glutamates is effectively constitutively protonated, since its pKa is below physiological pH. I think it would be useful to comment on this, given that the glutamates in the TPP bound simulations are both deprotonated.

   While it seems true that based on pKas from 10.1074/jbc.RA118.005430 one of the glutamates remains protonated in the cytoplasm-facing apo state, data from the same article indicates that ligand binding induces instantaneous proton release back to the cytoplasm (due to pKa being shifted by a charged ligand). Importantly, data in our Fig. 2A suggests that such a release is mechanistically feasible. Overall, while it might indeed add nuance to the order of events presented in Fig. 2C, it would not affect the overall mechanism of translocation. However, we acknowledge that a future study looking at the dependence of both glutamates' pKas as a function of z-distance would shed light on the details of this process.

9. Finally, the discussion around Figure 2 would be strengthened if it included discussion of alternative conformational exchange cycles for EmrE. It may be possible that the 2:1 stoichiometry is not a hard-and-fast rule (see 10.1073/pnas.1708671114, particularly its figure 5). If I read the order of events correctly, you have TPP binding first, followed by two protonation events. Clearly, the free energy change overall if all three of those had to happen each transport cycle would be the same. But what if, for instance, one proton remained bound during the full transport cycle? Would that be expected to be a faster process?

   If we follow the translocation pathway from Fig. 2 starting from the A-B0 state (olive), then a conformational swap brings the system to a deep near-midplane minimum in the A0B- state (dark green, since the conformational change swaps monomer labels) that doesn't feature an obvious way out except for immediate protonation of the other glutamate (mechanistically plausible but obviously pH- and pKa-dependent). The direct interpretation of the experimental setup from the PNAS article is further complicated by (a) the use of a different-shaped weaker-binding ligand (ethidium) and (b) introduction of transmembrane voltage. Moreover, the latter complication could work in non-linear ways, i.e. not only adding a constant slope to the profiles but also affecting the position of the deprotonated glutamates, and therefore changing the location and depth of the individual free energy minima.

   We nevertheless agree that future computational work should try to address these concerns more explicitly, e.g. comparing the translocation dynamics of different ligands and dissecting the effect of transmembrane potential on the mechanism of ligand translocation.
